# Misfolded Fate: A Case Report of Transthyretin Cardiac Amyloidosis in Ghana

**DOI:** 10.7759/cureus.96055

**Published:** 2025-11-04

**Authors:** Andrew S Dzebu

**Affiliations:** 1 Cardiothoracic Centre, Ho Teaching Hospital, Ho, GHA

**Keywords:** attr amyloidosis, cardiac scintigraphy, heart failure with preserved ejection fraction, infiltrative cardiomyopathy, tafamidis

## Abstract

Cardiac amyloidosis (CA), an entity once considered rare, is increasingly being diagnosed due to increasing awareness, more accessible diagnostic methods, and the recently available disease-modifying therapy. An 85-year-old woman with a history of long-standing hypertension, which has been well controlled, is diagnosed with heart failure with preserved ejection fraction. The 12-lead electrocardiogram showed red flags for amyloidosis. Transthoracic echocardiography showed unexplained biventricular hypertrophy. Cardiac scintigraphy showed a Perugini score of grade 3, consistent with cardiac amyloidosis. Serum free light chain ratio was normal, and monoclonal bands were absent on the electrophoresis of serum proteins. Genetic testing was not done due to limitations. The patient was treated with a sodium-glucose cotransporter-2 inhibitor (SGLT2i) and a mineralocorticoid receptor antagonist (MRA), without disease-modifying therapy such as tafamidis. This case highlights cardiac amyloidosis in a female patient and the feasibility of the non-biopsy pathway for diagnosing transthyretin cardiac amyloidosis in Ghana and across the broader African region. This condition is significantly underrecognized in the sub-region, despite the expectation that genetic variants are common.

## Introduction

Amyloidosis, once considered a rare disease, is increasingly being diagnosed partly due to increased awareness and improved availability of the appropriate technology. Over 30 types of amyloid proteins exist; however, the most clinically common ones, light chain (AL) and transthyretin (ATTR), are most prevalent, especially in developed countries, where almost all cases of amyloidosis are reported. These two types of amyloid tend to deposit in cardiac structures, leading to cardiac amyloidosis (CA) [1].

In the Ghanaian and broader sub-Saharan African (SSA) context, there is a scarcity of data on CA. Life expectancy has been increasing progressively on the continent, and a significant proportion of the population falls within the age range where wild-type transthyretin amyloidosis (ATTRwt) is more prevalent [2]. Furthermore, genetic variants of ATTR (ATTRv), specifically Val142Ile, are known to be common among people of African descent, especially in West Africa [3]. Is it, therefore, safe to assume that CA is underdiagnosed in SSA?

We present an 85-year-old woman with a history of long-standing essential hypertension, which has been well controlled, who eventually progressively developed recurrent heart failure with preserved ejection fraction.

## Case presentation

We present an 85-year-old black female with a 20-year history of essential hypertension, well-controlled on amlodipine 10 mg daily. She occasionally experiences episodes of dizziness associated with relatively low blood pressures (100-110/60-70 mmHg). Two years ago, she presented to her local hospital with a complaint of dyspnea on activities of moderate intensity. She was evaluated, and furosemide was added to her treatment. She mentions that this improved her symptoms for several months. However, throughout the latter end of the ensuing year, symptoms recurred even with activities of mild intensity. This time, she was also coughing, could not tolerate the decubitus position, and had notable swelling in her feet.

She was referred to a regional hospital, where she was assessed and diagnosed with hypertensive heart disease with heart failure. She recalls being confused about the diagnosis, knowing that her blood pressure had been well controlled from the time she was diagnosed with essential hypertension and placed on treatment. She also recalls that she did not tolerate anti-failure medications well, due to hypotension. She has been admitted several times for decompensated heart failure.

**Figure 1:**
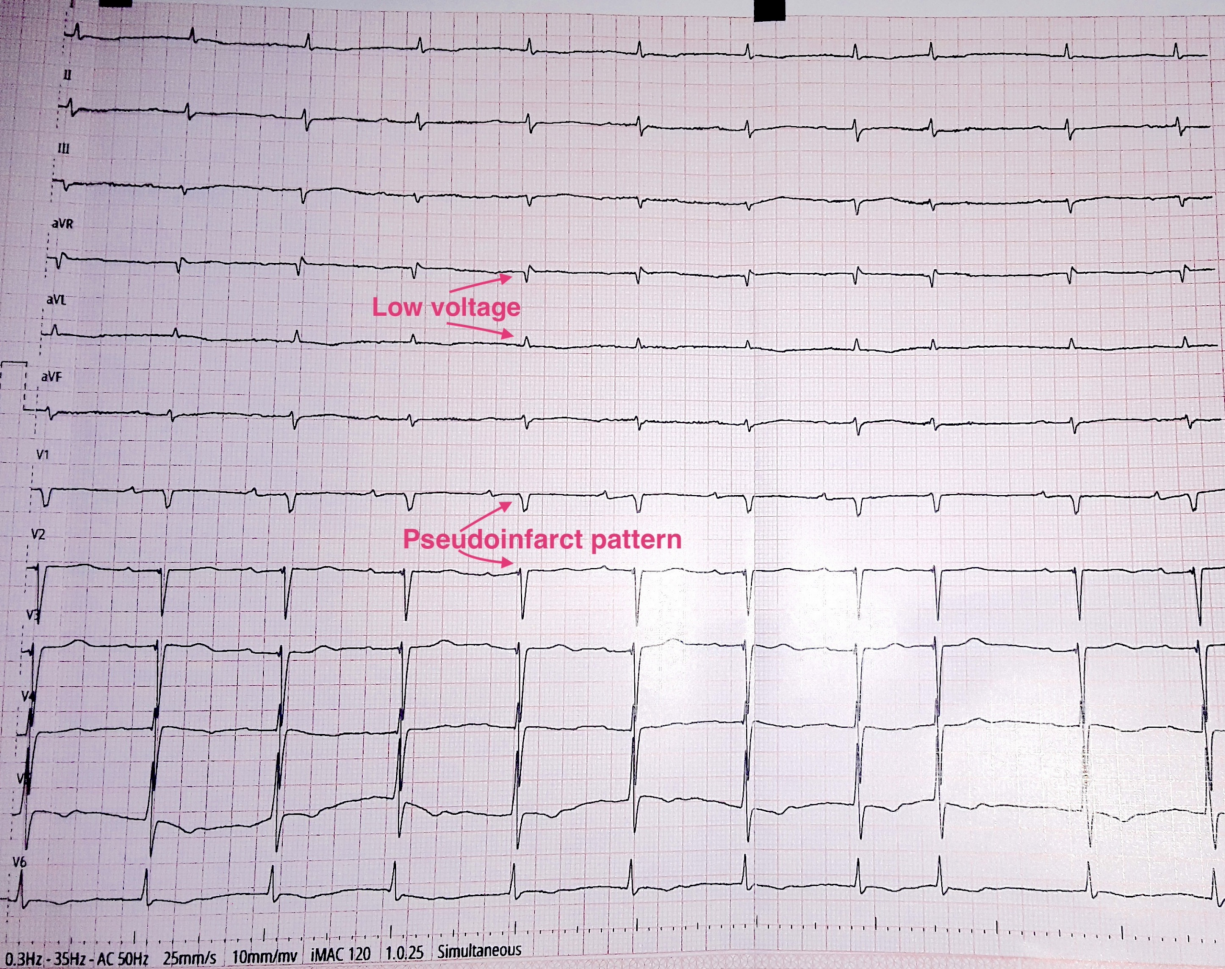
12-lead electrocardiogram showing red flags for amyloidosis Voltage criteria for hypertrophy (such as Sokolow-Lyon index for the left ventricle) are not met, at variance with the extent of "hypertrophy" observed on 2D echocardiography.

**Figure 2:**
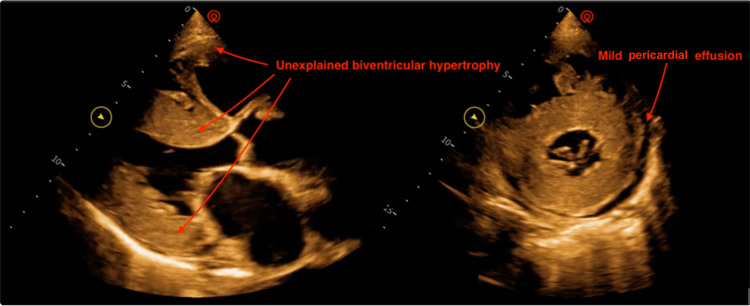
Transthoracic echocardiography showing unexplained biventricular hypertrophy with mild pericardial effusion and mildly thickened valve leaflets

The patient was referred to a tertiary hospital for further management. History was consistent with recurrent episodes of heart failure as expounded. A 12-lead electrocardiography revealed a sinus rhythm with left-axis deviation, low voltages, and a pseudo-infarct pattern in the septal leads (Figure 1). During transthoracic echocardiography, the findings were suggestive of severe biventricular hypertrophy, with grade 2 diastolic dysfunction and a high probability of pulmonary hypertension (Figure 2). Recommendations for multimodality imaging were made to further investigate this patient; however, they could not be implemented due to limited funds.

**Figure 3:**
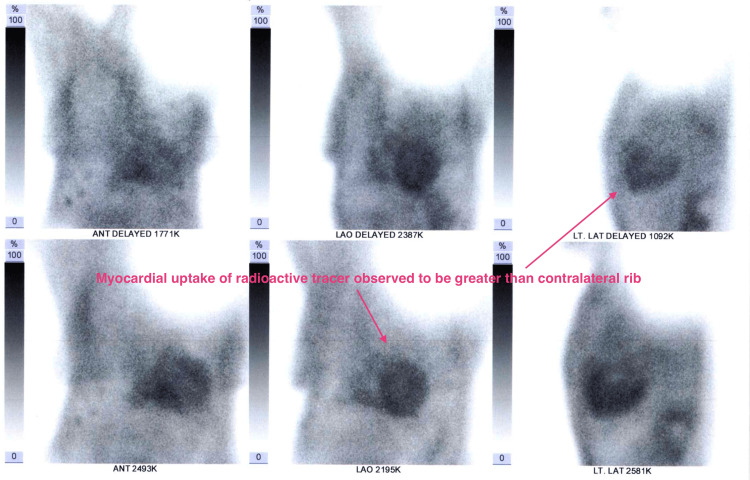
Cardiac scintigraphy showing increased myocardial uptake of the radioactive tracer ANT: anterior, LAO: left anterior oblique, LT LAT: left lateral

**Figure 4:**
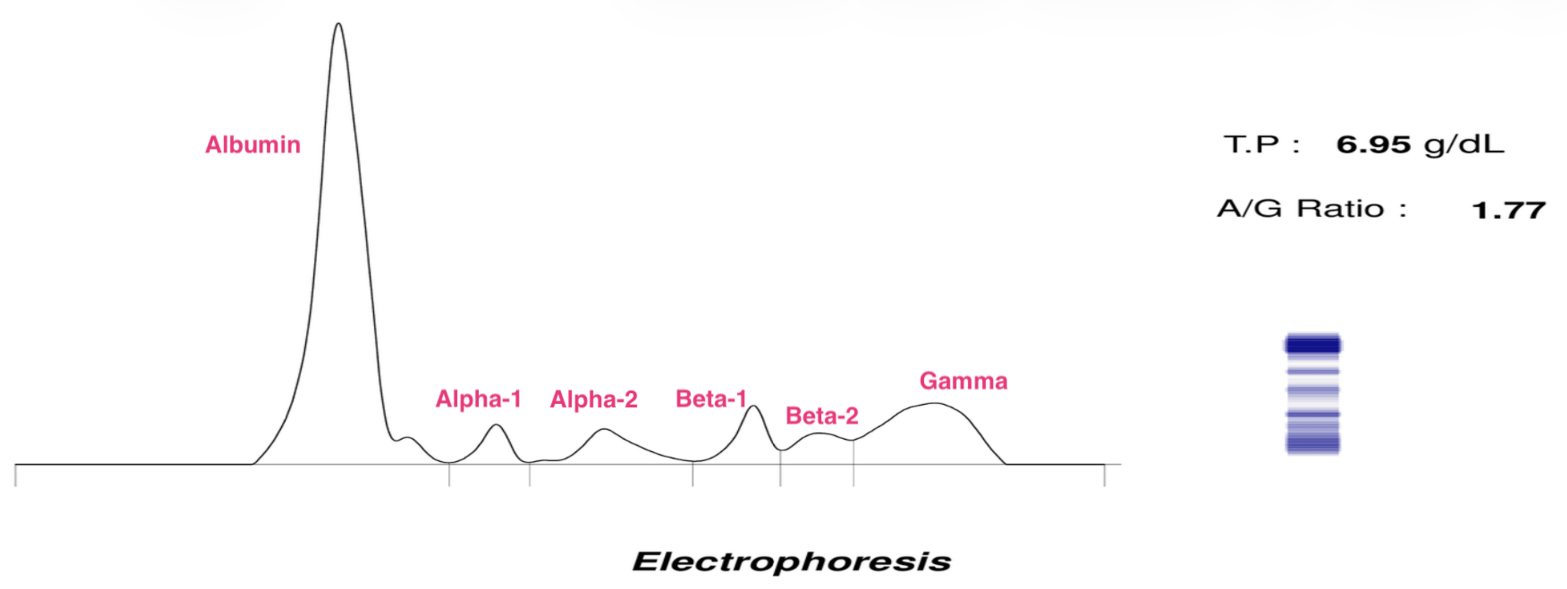
Serum protein electrophoresis showing an absent monoclonal band T.P: total proteins, A/G: albumin/globulin ratio

Cardiac scintigraphy was significant for a grade 3 Perugini score, consistent with cardiac amyloidosis (Figure 3). Serum free kappa/lambda ratio was normal. Serum protein electrophoresis was insignificant for a monoclonal band (Figure 4, Table 1). It was therefore concluded that the patient had ATTR-CA. Genetic testing, not available in-country, but procurable abroad, comes at a prohibitive cost and therefore cannot be done.

**Table 1:** Relative values of serum proteins assessed by electrophoresis

Fractions	Percentage	Reference range (%)
Albumin	63.9	55.8-66.1
Alpha 1	3.4	2.9-4.9
Alpha 2	6	7.1-11.8
Beta 1	5.7	4.7-7.2
Beta 2	4.8	3.2-6.5
Gamma	16.2	11.1-18.8

Disease-modifying treatments, such as tafamidis and acoramidis, are all unavailable in-country. The patient was therefore only placed on symptomatic therapy for heart failure with preserved ejection fraction: empagliflozin per os 10 mg once daily, spironolactone per os 25 mg once daily, and diuretics such as furosemide tailored according to the presence and severity of congestions.

The patient has been grossly asymptomatic for about three months, but it is expected that she will relapse and the disease will progress due to the absence of disease-modifying therapy.

## Discussion

Amyloidosis is a systemic disease characterized by the extracellular deposition of misfolded proteins in various organs [1,4]. CA, once thought to be a rare cause of heart failure, has now been increasingly identified as a cause of heart failure. The pathological basis of ATTR is due to the infiltration of monomers of transthyretin (a protein that transports thyroxine and retinol) in the cardiac extracellular space. This leads to a functionally restrictive cardiomyopathy, usually with "hypertrophy" [3]. TTR in the cardiac interstitium can be cytotoxic to cardiomyocytes (leading to inflammation and fibrosis), which may contribute to diastolic dysfunction, arrhythmogenesis, and thrombogenesis. TTR infiltration can also cause mitochondrial dysfunction and oxidative stress, and disrupt calcium levels and handling [5]. In most cases, more than 98% of CA are ATTR or AL. ATTR-CA can be either acquired (ATTRwt) or due to rare genetic causes (ATTRv) [1].

Black populations are at particular risk, given that a gene responsible for hereditary ATTR is known to be prevalent among people of West African ancestry [3]. ATTRwt shows a strong male predominance, with a male-to-female ratio of 25-50:1 [4]. Also, the most commonly diagnosed myocardial disease in Africa is usually hypertensive heart disease [6]. This may be due partly to a confounding high burden of hypertension in the general population [7], low awareness and therefore less consideration of infiltrative and other myocardial disease in differential diagnosis, and lack of physical and/or financial access to multimodality imaging technology such as echocardiography with strain imaging, cardiac magnetic resonance imaging (MRI), scintigraphy, and computed tomography (CT) [3]. A few pathological series on cardiomegaly conducted in Ghana relied solely on macroscopic examination and therefore could not adequately characterize the disease [6].

The diagnosis of CA should be entertained in the presence of "red flags," including those listed in the European Society of Cardiology position statement. These could be divided into clinical (hypotension/normotension in a previously hypertensive patient, polyneuropathy, carpal tunnel syndrome, etc.), electrocardiographic (low voltage, pseudo-infarct pattern, atrioventricular conduction disease, etc.), and echocardiographic/MRI-related (biventricular hypertrophy, increased valve thickness, apical sparring pattern on strain imaging, etc.) [1,8]. The patient in question exhibited several red flags, including electrocardiographic elements.

Having identified the described reg flags, it is appropriate to confirm the diagnosis of cardiac amyloidosis using an invasive biopsy (examined using Congo red staining) or a non-biopsy pathway. Endomyocardial biopsies can be obtained in a few centers with experience in the procedure, which, to the best of our knowledge, is limited or unavailable in sub-Saharan Africa. A biopsy can also be obtained from surrogate tissue, which may also be infiltrated by amyloid fibrils, given that amyloidosis is a systemic disease. The diagnostic yield of surrogate biopsies is inferior to that of endomyocardial biopsies. However, nine patients have been identified using abdominal fat pad biopsies in-country, with features highly suggestive of amyloid infiltration [9]. The non-biopsy pathway involves cardiac scintigraphy with bone-avid tracers such as 99mTc-diphosphono-1,2-propanodicarboxylic acid (99mTc-DPD), 99mTc-pyrophosphate (99mTc-PYP), or 99mTc-hydroxymethylene diphosphonate (99mTc-HMDP). Increased myocardial uptake, as determined by qualitative visual scoring (Perugini score 2 or 3), in the absence of detectable monoclonal proteins, is highly sensitive (99%) and specific (86%) for ATTR [10,11]. These conditions were met in our patient, particularly with the recent availability of cardiac scintigraphy.

Ghana, a country with a population exceeding 30 million people, has approximately 30 practicing cardiologists. One or two centers offer echocardiography with strain imaging, cardiac CT, MRI, and scintigraphy, all of which are located in the capital city of Accra. However, access to these services is complicated by the need for patients to make out-of-pocket payments, which are often beyond the means of the average worker. A combination of these factors has led to the systematic underdiagnosis of cardiac amyloidosis.

Additionally, disease-modifying therapy for ATTR is unavailable in Ghana and, to a greater extent, likely in the SSA region. These therapies include TTR stabilizers (e.g., tafamidis and acoramidis) and synthesis suppressors using gene editing (e.g., Patisiran and Vutrisiran). Drugs that facilitate the elimination of amyloid fibrils, such as doxycycline and green tea, have not been evaluated in clinical trials. Disease-modifying therapy must be initiated early for optimal mortality benefits [1,3]. Unfortunately, this patient and those diagnosed previously still do not have access to disease-modifying therapy. The use of sodium-glucose cotransporter-2 inhibitors (SGLT2i) has been shown to reduce a composite of all-cause mortality and heart failure hospitalizations in patients with CA-ATTR [12]. Mineralocorticoid receptor antagonists (MRAs) are well tolerated by patients with ATTR-CA and are associated with mortality benefits [13]. Patients with AL CA may be treated with standard chemotherapy according to the kind of plasma cell disorder they are diagnosed with [1].

Importantly, increased awareness among all stakeholders in the healthcare ecosystem is required for an international effort to ensure timely diagnosis and treatment of cardiac amyloidosis.

## Conclusions

Cardiac amyloidosis, once thought to be rare, is increasingly being diagnosed as a cause of heart failure. Limited awareness of diagnostic technologies in the Ghanaian and larger sub-Saharan African context has largely hindered its diagnosis. Non-biopsy diagnosis, such as myocardial radiotracer uptake evaluated by cardiac scintigraphy in the absence of monoclonal proteins, is an acceptable method in limited-resource settings like Ghana, where endomyocardial biopsy is not performed. Recently approved effective therapies, such as tafamidis, have greatly intensified the need for early diagnosis and treatment to improve outcomes. However, they are unavailable in Ghana and most of sub-Saharan Africa. SGLT2i and MRA are essential additional therapies for patients with ATTR-CA. This case also uniquely highlights a female patient with ATTR-CA, a disease that primarily affects men.
